# Best Practices in Developing a Workflow for Uncertainty Quantification for Modeling the Biodegradation of Mg‐Based Implants

**DOI:** 10.1002/advs.202403543

**Published:** 2024-10-25

**Authors:** Tamadur AlBaraghtheh, Regine Willumeit‐Römer, Berit Zeller‐Plumhoff

**Affiliations:** ^1^ Institute of Metallic Biomaterials Helmholtz‐Zentrum hereon GmbH Max‐Planck‐Straße 1 21502 Geesthacht Germany; ^2^ Institute of Surface Science Helmholtz‐Zentrum hereon GmbH Max‐Planck‐Straße 1 21502 Geesthacht Germany; ^3^ Data‐driven Analysis and Design of Materials Mechanical Engineering and Marine Technologies University of Rostock Albert‐Einstein‐Straße 2 18059 Rostock Germany

**Keywords:** biodegradable Mg‐implants, propagation, surrogate model, uncertainty, UQ

## Abstract

Computational models of electrochemical biodegradation of magnesium (Mg)‐based implants are uncertain. To quantify the model uncertainty, iterative evaluations are needed. This presents a challenge, especially for complex, multiscale models as is the case here. Approximating high‐cost and complex models with easy‐to‐evaluate surrogate models can reduce the computational burden. However, the application of this approach to complex degradation models remains limited and understudied. This work provides a workflow to quantify different types of uncertainty within biodegradation models. Three surrogate models—Kriging, polynomial chaos expansion, and polynomial chaos Kriging—are compared based on the minimum number of samples required for surrogate model construction, surrogate model accuracy, and computational time. The surrogate models are tested for three computational models representing Mg‐based implant biodegradation. Global sensitivity analysis and uncertainty propagation are used to analyze the uncertainties associated with the different models. The findings indicate that Kriging proves effective for calibrating diverse computational models with minimal computational time and cost, while polynomial chaos expansion and polynomial chaos Kriging exhibit greater capability in predicting propagated uncertainties within the computational models.

## Graphical abstract

This study presents an Uncertainty Quantification (UQ) workflow for biodegradation models of magnesium‐based implants. It compares Kriging, polynomial chaos expansion (PCE), and polynomial chaos Kriging (PCK) for surrogate model selection, emphasizing computational efficiency, model accuracy, and uncertainty prediction. The workflow is scalable and adaptable, offering a robust framework for quantifying uncertainty in complex degradation systems.

UQ‐workflow of biodegradation models using surrogate models.

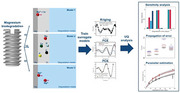

## 1 Introduction

Magnesium (Mg) and its alloys are being investigated increasingly as temporary bone implant materials due to their non‐toxicity, biocompatibility, and biodegradability.^[^ ^1^ ^]^ However, numerous in vitro and in vivo experiments are needed to tailor the degradation rates of these implants to be suitable for the human body.^[^ ^2^, ^3^ ^]^ A growing interest in digital twins, which use computational models to imitate and predict these experiments, has been stimulated by the increasing digitization of data and processes.^[^ ^4^, ^5^, ^6^ ^]^ As such, digital twins are expected to bridge the gap between the in vitro and in vivo experiments, while also reducing costs, experimental time, and the number of animal tests.^[^ ^7^, ^8^ ^]^

Degradation models, which will form the basis for the digital twin, possess a multi‐dimensional and multi‐scale nature, thereby resulting in a substantial computational load during their solution.^[^ ^9^, ^10^, ^11^ ^]^ Moreover, these models are inherently uncertain. While state‐of‐the‐art degradation models include a wide variety of complexities and can be used for certain applications, these models cannot be generalized to estimate the degradation of implants under different environmental conditions.^[^ ^9^, ^11^, ^12^, ^13^ ^]^ Uncertainty is an inherent characteristic of complex systems in mathematical models. This uncertainty arises from multiple sources, including uncertain input parameters, mathematical approximations, and the assumptions and hypotheses used to construct the model.^[^ ^14^ ^]^ Furthermore, limitations in computing resources contribute to these uncertainties by constraining numerical computation precision, the number of feasible model evaluations, and the scope of computations available for supporting analyses.^[^ ^15^, ^16^ ^]^ It is important to note that the reliability of a degradation model is influenced both by the uncertainty associated with the estimations of the computational model and the uncertainty associated with the observations of the physical system, which must be quantified in order to build a digital twin of in vivo and in vitro experiments.^[^ ^4^, ^6^, ^8^, ^13^ ^]^

In uncertainty quantification (UQ), models are analyzed within an uncertainty framework that assesses and quantifies the impact of uncertainties on model predictions. Such analysis involves assessing the impacts of uncertainty on reliability and robustness, calibration using experimental data, or conducting global sensitivity analysis.^[^ ^13^, ^17^ ^]^ Two main approaches exist for UQ techniques: intrusive and non‐intrusive techniques. The intrusive approach involves incorporating uncertain variables into the governing equations of the mathematical model and modifying the source codes of the numerical models.^[^ ^18^, ^19^, ^20^ ^]^ However, these modifications become challenging as the mathematical complexity of the computational model increases, leading to increased CPU usage and computational times.^[^ ^19^, ^21^ ^]^ Moreover, these techniques are not feasible when source codes are unavailable to end‐users, e.g., when using commercial software. On the other hand, non‐intrusive techniques treat the original model as a black box and rely on sampling techniques to explore the model's input space and evaluate the model's responses without altering its internal structure.^[^ ^19^, ^21^, ^22^, ^23^ ^]^ In general, the uncertainty inherent in the degradation models of Mg‐based implants can be assessed through non‐intrusive UQ methods. However, although non‐intrusive UQ techniques are straightforward from a methodological point of view, they are computationally demanding even with high‐performance computing resources.^[^ ^6^, ^19^ ^]^ To address this challenge, surrogate modeling is employed, wherein complex models are approximated by fast‐to‐evaluate analytical functions based on an input/output mapping of the original model.^[^ ^24^, ^25^ ^]^

In recent years, surrogate modeling has gained significant interest, as these models can be trained using limited data obtained from the complex multi‐scale model.^[^ ^24^, ^25^, ^26^ ^]^ This development in the approach has led to the creation of various classes of surrogate models; a detailed review can be found in ref. [25]. These classes are differentiated based on type, training strategies, data requirements and application‐specific criteria. Consequently, selecting the appropriate surrogate model for the system under study becomes crucial.^[^ ^6^, ^15^, ^27^, ^28^, ^29^ ^]^ A surrogate model's effectiveness and accuracy are determined by a combination of several factors, each of which plays an important role in its reliability and performance. Among these factors are the mathematical nature of the original model functions, the selection of a surrogate model type tailored to the specific domain of the problem, and its capability to enhance understanding of the model's input‐output relationships.^[^ ^30^ ^]^ One of the most influential factors affecting surrogate model performance is the quality and quantity of training data used during its construction, as well as the choice of the set of input variables and the control of the noise inherent in these variables.^[^ ^28^, ^31^ ^]^ Equally significant is the level of complexity of the surrogate model itself. Excessively simplified surrogate models might not have the capacity to capture the underlying behavior of the system, while an overly complex one risks overfitting and subsequent poor generalization to unseen data.^[^ ^6^, ^28^ ^]^ Additionally, accurately tuning the surrogate model's parameters is essential for achieving accuracy and high performance.^[^ ^24^ ^]^ Incorporating domain knowledge enhances the performance of surrogate models by providing a comprehensive understanding of the underlying phenomenon of the system. Also, proper pre‐processing of the data, coupled with the use of independent datasets to validate and test the surrogate models, can significantly boost their performance and accuracy.^[^ ^15^, ^27^, ^28^ ^]^ Furthermore, it is imperative to reconstruct the surrogate models to align with advancements in the original models they represent as well as keeping a balance between accuracy and computational efficiency is a crucial issue for optimal performance.^[^ ^15^, ^29^ ^]^

In simulating the degradation of biodegradable implants, surrogate models play a promising role. Therefore, the focus of this manuscript is to present a best‐practice workflow for constructing and applying such surrogate models to different degradation models. Although the demonstration of these methods is performed using simpler in vitro models, the proposed workflow is also applicable to more complex models. As we have demonstrated previously, under certain conditions, it is possible to utilize surrogate models to expand the parametric space and simulate in vivo corrosion based on in vitro corrosion.^[^ ^8^ ^]^ Furthermore, the paper assesses the impact of three surrogate modeling techniques, namely Kriging, Polynomial Chaos Expansion (PCE) and polynomial chaos Kriging (PCK) on the uncertainty analysis of the degradation models. These techniques are evaluated using three different mathematical models that characterize various aspects and phenomena related to the degradation of biodegradable Mg‐based alloys. The degradation models vary in terms of mathematical complexity, dimensionality and model construction hypotheses and assumptions. During the development of these surrogate models, we will examine the influence of parameter sampling strategies, including adaptive sampling. A further objective of the study is to test and analyze the performance of these surrogate models for two fundamental forward UQ tasks: sensitivity analysis and uncertainty propagation.

## 2 Experimental Section

### 2.1 Overview of the Degradation Process

The general concept of the degradation and the main factors affecting it have been presented in refs. [1, 13, 32]. As a summary, the degradation of Mg in aqueous media is an electrochemical reaction (Equation 1), which involves an anodic reaction for Mg (Equation 2), while water goes through a cathodic reaction (Equation 3). Ultimately, magnesium hydroxide and hydrogen gas (H_2_) are produced.

(1) $${\text{Mg}}_{\text{(s)}}+{2H_{2}O}_{\text{(aq)}}\to {\text{Mg}{\left(OH\right)}_{2}}_{\text{(s)}}+{H_{2}}_{\text{(g)}}\;\text{(over all reaction)}$$

(2) $${\text{Mg}}_{\text{(s)}}\to {\text{Mg}}_{\text{(aq)}}^{2+}+2e^{-}\;\text{(anodic reaction)}$$

(3) $${2H_{2}O}_{\text{(aq)}}+2e^{-}\to {H_{2}}_{\text{(g)}}+{\text{OH}}_{\text{(aq)}}^{-}\;\text{(cathodic reaction)}$$

Equation (3), one mole of Mg must be degraded in order to produce one mole of H_2_. While according to Equation (1), the overall degradation reaction depends on the degradation medium as a function of the pH.^[^ ^32^, ^33^ ^]^ It is anticipated that a passive layer of magnesium hydroxide, Mg(OH)_2_, will develop in response to an increasing pH value. This layer will act as a protective layer to increase the corrosion resistance of the metal. However, this layer is only stable when the pH level is highly alkaline (pH >11).^[^ ^32^, ^34^, ^35^ ^]^ A more stable thin, porous MgO layer (less than 10 nm) was observed to form due to interactions with air.^[^ ^36^ ^]^ Corrosion of metal is an electrochemical process that occurs when a metal interacts with an aqueous electrolyte and changes its oxidation state, as demonstrated by Equation (2). During this process, electrons are transferred between the electrode's interface and metal atoms are oxidized to form ionic species, with electrons being liberated at the same time.^[^ ^34^, ^37^ ^]^

Generally, degradation systems of biodegradable bone implants operate within a pH range of 7.4 ± 0.4,^[^ ^38^ ^]^ allowing the formation of several precipitates based on the ions present in the degradation medium. The chemical reactions of the precipitates in this study are presented in the following equations, with the name of each precipitate indicated alongside its corresponding equation.^[^ ^37^ ^]^ Note that the precipitation of Bobierrite, Equation (10), was not considered in any of the degradation models.

(4) $${\text{Mg}}^{2+}\text{(aq)}+2{\text{OH}}^{-}\text{(aq)}\to {\text{Mg(OH)}}_{2}\text{(s)}\text{(Brucite)}$$

(5) $${\text{Mg}}^{2+}\text{(aq)}+{\text{HCO}}_{3}^{-}\text{(aq)}\to {\text{MgCO}}_{3}\text{(s)}+H^{+}\text{(aq)}\text{(Magnesite)}$$

(6) $$\begin{array}{ccc} {\text{Mg}}^{2+}\text{(aq)} & & +{\text{HCO}}_{3}^{-}\text{(aq)}+3H_{2}O\to \text{Mg}\left({\text{HCO}}_{3}\right)\text{OH}\cdot 2H_{2}\text{O(s)}\\ & & +H^{+}\text{(aq)}\text{(Nesquehonite)} \end{array}$$

(7) $${\text{Ca}}^{2+}\text{(aq)}+2{\text{OH}}^{-}\text{(aq)}\to {\text{Ca(OH)}}_{2}\text{(s)}\text{(Portlandite)}$$

(8) $${\text{Ca}}^{2+}\text{(aq)}+{\text{HCO}}_{3}^{-}\text{(aq)}\to {\text{CaCO}}_{3}\text{(s)}+H^{+}\text{(aq)}\text{(Calcite)}$$

(9)

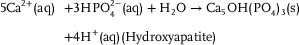

(10) $$\begin{array}{ccc} 3{\text{Mg}}^{2+}\text{(aq)} & & +2{\text{HPO}}_{4}^{2-}\text{(aq)}+8H_{2}O\to {\text{Mg}}_{3}{\left({\text{PO}}_{4}\right)}_{2}\cdot 8H_{2}\text{O(s)}\\ & & +2H^{+}\text{(aq)}\text{(Bobierrite)} \end{array}$$

#### 2.1.1 Numerical Simulation and Model Development of the Degradation Process

In the current study, three finite element models were conducted to simulate different aspects of the degradation behavior of Mg‐based implants.

**Figure 1:**
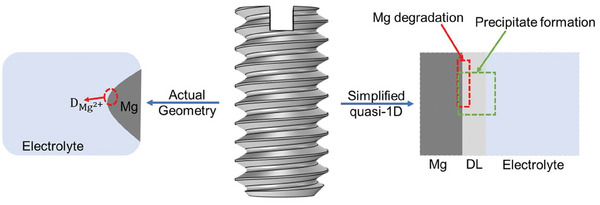
Schematic illustration of the approximated numerical geometries of Mg‐based implants for the three degradation models. To the right, the implant geometry is simplified using quasi‐1D geometry for both the degradation model of pure Mg and the formation of precipitate.^[^ ^39^ ^]^ Left schematic shows the focus on the diffusion of Mg^2 +^‐ions from the implant surface to the electrolyte.

The first model is a generalized quasi‐1D FE model of pure Mg degradation in simulated body fluid (SBF) over the course of 28 days, which was taken from ref. [39]. In the second FE model, a second aspect of the model derived in ref. [39] was considered, which is the simulation of the formation and precipitation of relevant degradation products on the surface of the implant over the course of 28 days. For the third FE model, a simplified 3D degradation model of an actual implant geometry based on diffusion was derived. For this model, experimental data of Mg‐xGd (Mg‐5 wt.% Gd and Mg‐10 wt.% Gd) over the course of 56 days were used for the calibration process. The schematic diagram in **Figure** **1** illustrates the models' basic principles and geometries.

In a brief summary of the degradation models, the generalized quasi‐1D model of pure Mg degradation in SBF, illustrated in Figure 1, marked with a red box on the right of the implant rendering, was derived based on experimental observations by refs. [40, 41]. In this model, Mg degradation was found to have an initial period of low degradation followed by a linear trend. The mathematical presentation of the effective rate of Mg^2+^ ions released at the metallic‐liquid interface is given by

(11) $$\frac{\partial c_{\mathrm{Mg}}}{\partial t}=\frac{\varepsilon k_{\deg}}{1+\exp^{-(t-t_{\mathrm{init}})}}$$

where ∂*c*_*Mg*_/∂*t* is the change in the concentration profile of Mg (*c*_*Mg*_) over time and *t*_*init*_ denotes the starting time of degradation, taking into account the initial thin degradation layer of MgO. ϵ and *k*_*deg*_ are the porosity of the degradation layer and the degradation rate constant, respectively. The dynamic deformation of the model geometry was captured using the arbitrary Lagrangian–Euler (ALE) method, which was utilized to explicitly track the moving interface of the degraded Mg.^[^ ^39^, ^42^, ^43^, ^44^ ^]^ The degradation of Mg was then calculated in terms of the mean degradation depth (MDD) as

(12) $$\mathrm{MDD}=r_{\mathrm{init}\mathrm{ial}}-\int v_{\deg}\mathrm{dt}$$

where *r*_*initial*_ is the length of the Mg‐domain and *v*_*deg*_ is the normal velocity of the metal interface and it is computed based on the concentration gradient of the Mg as

(13) $$v_{deg}=\frac{M_{Mg}}{\rho_{Mg}}\frac{\partial c_{Mg}}{\partial t}$$

where *M*_*Mg*_ and ρ_*Mg*_ are the molecular weight and the density of Mg, respectively.

The formation of precipitates on the surface of the implant from different chemical species in the medium, marked with a green box on the right of the implant rendering, is modeled using the Nernst–Planck Equation (NPE) (14). NPE is implemented to mathematically describe the transport of chemical species *i* of concentration *c*_*i*_ and it is given by

(14) $$\frac{\partial c_{i}}{\partial t}+\nabla \cdot \left(D_{i}\nabla c_{i}-z_{i}\frac{D_{i}}{\mathrm{RT}}Fc_{i}\nabla V\right)=R_{i}$$

The left‐hand side of the NPE represents the species flux, where the first part describes the diffusion of species within the porous degradation layer (DL) and the electrolyte. While the migration of species within the electric field is described in the second part of NPE (14). *D*_*i*_ denotes the diffusion coefficient of the chemical species in the liquid phase of the porous medium, *z*_*i*_ is the species charge, *V* is the electric potential, R ≈ 8.314 J (mol K)^−1^ is the gas constant and T is the temperature. F≈ 96485 C mol^−1^ is the Faraday constant. The right‐hand side of NPE, R_*i*_, is the chemical reaction rate of species *i*. The analysis of the degradation layer showed that it contained a number of different precipitates.^[^ ^38^ ^]^ Under the assumption that the formation of precipitates on Mg surface can only take place in the porous degradation layer, the formation of precipitate *p*_*l*_, according to the chemical Equations (4)–(9), can be mathematically modeled as^[^ ^39^ ^]^

(15) $$\frac{\partial c_{p_{l}}}{\partial t}=\varepsilon k_{l}\left(K_{\mathrm{eq},l}-c_{i}c_{j}\right)$$

where *K*_*eq*, *l*_ is the equilibrium constant and *k*_*l*_ is the backward reaction constant for reaction *l* of the Equations (4)–(9) and *i* and *j* denote the two species reacting in the respective reaction. Based on the concentration of each precipitated species $c_{p_{l}}$ the elemental wt% of each chemical element within the degradation layer as

(16) $$wt_{\mathrm{DL},\mathrm{ele}m_{i}}=\frac{\rho_{\mathrm{DL},\mathrm{ele}m_{i}}}{\sum_{k=1}^{6}\rho_{\mathrm{DL},\mathrm{ele}m_{k}}}\cdot 100$$

where *elem*_*i*_ stands for the chemical elements C, Ca, P, H, O, and Mg. $\rho_{DL,elem_{i}}$ is overall mass concentration in the degradation layer of element *i*.

The simplified 3D degradation model is derived from NPE (14) under the assumption that the diffusion of Mg^2+^ and other ions is the rate‐limiting step for degradation. Under the assumption that degradation is governed by the diffusion of Mg^2 +^ ions from the implant surface within the degradation medium.^[^ ^42^, ^43^, ^45^ ^]^ Under this assumption, the NPE (14) reduces into Fick's law^[^ ^46^ ^]^ and the diffusion of Mg^2+^ ions is modeled as

(17) $$\frac{\partial c_{\mathrm{Mg}}}{\partial t}=-\nabla \cdot \left(D_{\mathrm{Mg}}\nabla c_{\mathrm{Mg}}\right)$$

where *D*_*Mg*_ is the diffusion coefficient of Mg^2+^ ions from the implant surface into the degradation medium. In the study of Mg‐based alloys, diffusion‐only models assume the formation of a uniform corrosion layer on the alloy's surface, with degradation progressing uniformly as Mg^2+^ ions diffuse through this layer. However, these models face limitations in accurately capturing the non‐uniform degradation behavior observed in experimental settings. Despite this limitation, the application of diffusion‐only models to study alloy degradation remains valid. These models can adjust key parameters, such as the diffusion coefficient and degradation layer thickness, to align with experimental data. While these parameters may not directly reflect the alloy's heterogeneous microstructure and surface morphology, they still offer a macroscopic perspective on the degradation process.^[^ ^39^, ^47^ ^]^ The quasi‐1D FE model of pure Mg degradation in SBF, which simulates degradation and precipitate formation, is discretized using rectangular FE and implemented in COMSOL Multiphysics 6.2 (COMSOL AB, Stockholm, Sweden). A symmetric quasi‐1D geometry with a height of 1 µm and equal domains of 0.2 mm for both the Mg and electrolyte domains is employed. Additionally, an initial degradation layer of 10 nm is considered, utilizing a variable thickness mesh that is refined near the degradation layer.^[^ ^39^ ^]^ For the simplified 3D degradation model, a realistic 3D screw identical to the geometry of the experimental screws used in ref. [48] is used. A CAD mesh is imported into COMSOL Multiphysics 6.2, then a free tetrahedral mesh of the implant, which measures 4 mm in length, 2 mm in diameter with a M2 thread and a 0.5 × 0.5 mm slotted screw head, is created surrounded by the geometry of the electrolyte, which has a dimension of 3x3x5 mm. The mesh is smoothed through four iterations and it is bound by 0.03 and 0.05 mm to avoid staircase effects. Zero initial concentration of Mg^2+^ ions within the electrolyte is assumed. A constant diffusion coefficient of the chemical species in the liquid phase of the porous medium is set to equal 1.4 × 10^−16^ m^2^ s^−1^.

The three biodegradation models mentioned above were selected to ensure the flexibility and generalization of the proposed UQ‐workflow. Although these models may seem simple from a computational and mathematical standpoint, their straightforward nature is intentional. It highlights the surrogate model's ability to be tailored to the unique characteristics of different degradation systems without unnecessary complexity. Furthermore, by employing models with varying levels of complexity, we effectively harness the strengths of both simple and intricate surrogate models, ensuring that our approach remains broadly applicable and generalizable to more complex degradation scenarios.

#### 2.1.2 Calibration Data

The degradation models are calibrated against in‐house experimental data. The calibration date for the generalized quasi‐1D FE model of pure Mg degradation is obtained by evaluating the mean degradation depth of the Mg specimen using both data from µCT imaging and weight loss data. The calibration data were taken from the original model publication.^[^ ^39^ ^]^ The formation of precipitates data is obtained from EDX measurements, originally published in refs. [40, 49], in which the mean elemental weight percentage (wt%) of each element in the degradation layer is calculated. For both models a 28‐day degradation period is covered by experimental data. The simplified 3D degradation model is calibrated using volume loss (VL in %) data of Mg‐5 wt.% Gd and Mg‐10 wt.% Gd, which is obtained from in vitro tests conducted and published in ref. [50]. The volume loss % is measuring the difference in the implant volume over the degradation time, within the degradation model. The volume loss is measured as a function of the concentration changed along the degradation time.Since the concentration of Mg can be expressed as a function of number of Mg moles (N_*Mg*_) and the volume of Mg within the implant *c*_*Mg*_ = *N*_*Mg*_/*V*_*Mg*_, Equation (17) can be written as:

(18) $$\frac{\partial (\rho_{Mg}.V_{Mg})}{\partial t}=-\nabla \cdot \left(D_{Mg}\nabla \left(\rho_{Mg}.V_{Mg}\right)\right)$$

where ρ_*Mg*_ is the density of Mg within the implant, which is 1.81 and 1.87/g cm^−3^ for Mg‐5 wt.% Gd and Mg‐10 wt.% Gd, respectively. V_*Mg*_ is the volume of Mg at time t, based on Equation (18), the volume loss % is calculated as:

(19) $$VL\left(t\right)=\frac{V(0)-V(t)}{V(0)}\cdot 100\%$$

where V(0) is the initial volume of the implant and V(t) is the residual volume calculated by Equation (18). Note that the initial concentrations of Mg within the implants are adapted from the experiment; 7.13 × 10^−5^ and 7.07 × 10^−5^ mol mm^−3^ for Mg‐5 wt.% Gd and Mg‐10 wt.% Gd, respectively. The degradation rate (DR) in mm yr^−1^ is calculated based on volume loss as

(20) $$DR\left(t\right)=\frac{V(0)-V(t)}{A(0)\cdot t}$$

where *A*(0) is the initial surface area of the sample and *t* is the degradation time.

### 2.2 Overview of Surrogate Modeling

Several surrogate models have been developed and tested for different engineering problems. However, there is no consensus or clear‐cut guidelines on the selection process in the literature.^[^ ^30^ ^]^ In the present study, the focus was on surrogate models that treat the computational model as a black box which means these models only need the input values of model parameters and the corresponding output values of the quantity of interest (QoI). Here, a comparative analysis of three surrogate models was conducted, specifically Kriging (Krg), Polynomial Chaos Expansion (PCE), and Polynomial Chaos Kriging (PCK). Their performance and capacity were assessed to accurately represent degradation behavior. These surrogate models are evaluated throughout three specific tasks: parameter estimation, prediction of the original model's outcomes and sensitivity analysis within the context of degradation models. A general overview of surrogate models is provided in this section, including their construction and mathematical presentation.

The general mathematical format of the surrogate models is

(21) $${Y=\;M}^{S}\left(X\right)$$

where *S* represents the surrogate model method, in the current mathematical presentations *S* will be substituted with *Krg*, *PCE* and *PCK* for three different techniques. Y is the QoI for which the models are solved, M is the surrogate shape/mapping function, which can be a system of equations, a code or any corresponding functions that can be used to calculate Y values for any input parameters vector X for a finite period of time. Y and X for the current test cases are given in **Table** **1**.

**Table 1:** The surrogate model presentation of the QoI (Y) and the input parameter vector (X) for the three case studies of the degradation models of Mg‐based alloys.

Degradation model	X	Y
quasi‐1D degradation model of pure Mg	*k*_*deg*_, *t*_*init*_	*MDD*
Formation of precipitation on implant surface	*k*_*deg*_, *t*_*init*_, *k*_1_, *k*_2_, *k*_3_, *k*_4_, *k*_5_, *k*_6_, *k*_7_, *k*_8_	*wt*_*DL*, *Mg*_, *wt*_*DL*, *O*_, *wt*_*DL*, *P*_, *wt*_*DL*, *C*_, *wt*_*DL*, *Ca*_
3D degradation model of Mg‐alloy	*D*_*Mg*_	VL
where: *k*_*deg*_ is the degradation rate constant, *t*_*init*_ is the starting time of degradation/days, the reaction rate constants *k*_1_, *k*_2_, *k*_3_, *k*_4_, *k*_5_, *k*_6_, *k*_7_, and *k*_8_ correspond to the precipitation reactions represented by Equations (4)–(9), *D*_*Mg*_ = diffusion coefficient of Mg^2 +^ ions, *MDD* = mean degradation depth, *wt* = weight percentage of elements, where DL denotes the degradation layer and Mg, Ca, O, P, and C are the chemical elements in DL. VL is the volume loss		

#### 2.2.1 A Brief Review Surrogate Modeling Techniques

In this section a brief description of the three surrogate modeling techniques implemented in this study is presented.

##### Polynomial Chaos Expansion

The main idea of PCE is to expand the model response onto multivariate polynomial bases that are orthogonal to joint distributions of input variables. Consequently, evaluating the PCE coefficients is equivalent to characterizing the probability density function of the model response.^[^ ^25^, ^51^ ^]^ The QoI of the original model can be approximated by a finite, truncated set of polynomials

(22) $$Y=M^{PCE}\left(X\right)=\sum_{\underset{0\leq |\alpha |\leq p}{\alpha \in N^{M}}}y_{\alpha }\Psi_{\alpha }\left(X\right)$$

where $\Psi_{\alpha }\left(X\right)$ are multivariate polynomials orthonormal with respect to the joint probability density function f(X). $y_{\alpha }\in R$ are the expansion coefficients of the multivariate polynomials, which is estimated by a least‐square minimization method, and $\alpha \in N^{M}$ is a multi‐index that identifies the components of the multivariate polynomials Ψ_α_. The product of univariate polynomials is used to evaluate and build the multivariate polynomials as $\Psi_{\alpha }\left(X\right)=\prod_{i=1}^{n}\Psi_{\alpha_{i}}^{\left(i\right)}\left(X_{i}\right)$,^[^ ^52^, ^53^ ^]^ where $\Psi_{\alpha }^{\left(i\right)}$ is the polynomial in the *i* − *th* variable of degree α_*i*_. The total degree of multivariate polynomials is defined as $|\;\;\alpha \;\;|=\sum_{i=1}^{n}\alpha_{i}$. The total number of the expansion terms for the summation is ((*p* + *n*)!/*p*!*n*!), which is depending on the adopted truncation scheme.^[^ ^52^, ^53^ ^]^ Here, the hyperbolic index set based on q‐norms is implemented, where $\alpha \in N:||\alpha |{|}_{q}\leq p$ and *p* is the maximal total polynomial degree and the norm ∣∣α∣∣_*q*_ ⩽ *p* is defined as $||\alpha |{|}_{q}={\left(\sum_{i=1}^{n}\alpha_{i}^{q}\right)}^{1/q}$.^[^ ^52^ ^]^ The basis function of PCE can be described by several types of the orthogonal polynomials, e.g., Hermite, Laguerre, Jacobi, Legendre, Krawtchouk, Meixner, and Hahn.^[^ ^25^ ^]^ In the current study, a PCE degree‐adaptive calculation of the PCE coefficients, in particular the degree of polynomials, is implemented using the algorithm developed by ref. [54]. In other words, if an array of a specific range of possible degrees is given, the degree with the lowest ε_*LOO*_ error is selected automatically.

##### Kriging

The main idea behind Kriging, which is also known as Gaussian processing (GP), is to find the best linear unbiased prediction by minimizing the mean square error of the predictions.^[^ ^25^, ^55^ ^]^ The Kriging predictor, Equation (23), consists of two main parts: the deterministic polynomial term, which describes the global trend of the data. While the second part is the realization of a stochastic process that accounts for the lack of fit in the first part.^[^ ^30^ ^]^

(23) $$Y=M^{Krg}\left(X\right)=\beta^{T}f\left(X\right)+\sigma^{2}Z\left(X,\;\omega \right)$$

where $\beta^{T}f\left(X\right)$ is the mean value (trend) of GP, f(X) are arbitrary functions *f*_*j*_; *j* = 1, …, *P* that define the trend of the mean prediction at location X and β is the corresponding vector of unknown regression coefficients β_*j*_; *j* = 1, …, *P*, it should be selected precisely and it specifies the type of Kriging.^[^ ^30^, ^56^ ^]^ σ^2^ is the constant variance of GP and Z(X,ω) is a zero‐mean unit variance, stationary to the Gaussian process. ω is the probability space that is defined by a correlation function *R*(*x*_*i*_, *x*_*j*_, θ) and its hyperparameter θ, which is estimated by solving an optimization problem.^[^ ^30^ ^]^

The stochastic part of the Kriging equation requires the selection of the kernel, also known as the correlation function. The kernel describes the correlation between two sample points *x*_*i*_, *x*_*j*_ in the output space. In the current study the Mate'r 5/2 kernel, Equation (24), is used.^[^ ^57^ ^]^

(24) $$\begin{array}{ccc} R(x,x^{\prime },\nu =5/2) & & =\left(1+\sqrt{5}\frac{|x-x^{\prime }|}{\theta }+\frac{5}{3}{\left(\frac{|x-x^{\prime }|}{\theta }\right)}^{2}\right)\\ & & \times \exp\left[-\sqrt{5}\frac{|x-x^{\prime }|}{\theta }\right] \end{array}$$

The Matérn 5/2 kernel functions offer multiple hyperparameters, providing enhanced flexibility when fitting complex mathematical functions. Furthermore, the differentiability of the Kriging model depends on the smoothness of the kernel.^[^ ^58^, ^59^ ^]^ Smooth functions like Gaussian kernels produce very smooth interpolations, while the Matérn family, which is less smooth, can better capture non‐smooth functions.^[^ ^59^, ^60^ ^]^ Additionally, anisotropic kernels can model functions with varying degrees of importance across input variables because they have different length scales along different input dimensions.^[^ ^60^, ^61^ ^]^ Moreover, faster evaluations are ensured with fewer non‐zero coefficient Kriging models, which can be achieved by constructing sparse Kriging models, a capability provided by Matérn kernels.^[^ ^61^ ^]^ The characteristics of this kernel family enabled them to successfully fit all tested degradation models.

##### Polynomial Chaos Kriging

The PCK technique combines the two previous approaches in a simple way, leveraging each technique's strength.^[^ ^62^ ^]^ The PCK identifies the most relevant basis functions of model response adaptively. Moreover, it uses PCE as a regression‐based approach to capture global behavior of the computational model and uses Kriging as an interpolation‐based technique to capture local variation.^[^ ^63^ ^]^ In this sense, PCK can be seen as a universal Kriging model with a specific trend described by PCE.^[^ ^25^, ^55^ ^]^ The general formula of PCK is^[^ ^55^ ^]^

(25) $$Y=M^{PCK}\left(X\right)=\sum_{\underset{0\leq |\alpha |\leq p}{\alpha \in N^{M}}}y_{\alpha }\Psi_{\alpha }\left(X\right)+\sigma^{2}Z\left(X,\omega \right)$$

The PCK surrogate model was constructed sequentially, i.e., the set of optimal polynomials is found first and then the PCK model is calibrated to find the parameters of Kriging. This results in lower computational costs for the degree‐adaptive calculation of the PCE coefficients.

#### 2.2.2 Surrogate Models Performance

The surrogate models in this study are constructed using *k*‐fold cross validation (CV).^[^ ^25^, ^64^, ^65^, ^66^ ^]^ In CV, the training data is randomly divided into *k* equal sized subsets. One of these subsets is considered the validation set and it is used for model assessment, while all other sets are used as training sets, which helps avoid both underfitting and overfitting of the training data.^[^ ^65^, ^67^, ^68^ ^]^ Moreover, CV helps assess the generalization of the constructed surrogate models. This is because CV involves repeatedly training the surrogate models on subsets of the data and evaluating their performance on the remaining held‐out folds. The consistent performance of the surrogate models across the CV folds confirms their robustness and generalization capabilities. In particular, this means that a surrogate model is built *k* times, and its accuracy is measured by the total error in the predictions from each of these models, specifically by calculating the leave‐one‐out error (ε_*LOO*_) for each sample of size N.^[^ ^15^, ^64^, ^69^ ^]^

(26) $$\varepsilon_{LOO}=\frac{1}{N_{K}}\left[\frac{\sum_{i=1}^{N_{K}}{\left(M\left(x_{i}\right)-M_{\left(-i\right)}\left(x_{i}\right)\right)}^{2}}{Var\left[M(X)\right]}\right]$$

where *N* is the number of data points considered during building the surrogate model, $M\left(X_{i}\right)-M_{\left(-i\right)}\left(X_{i}\right)$ is the difference in prediction between the original observation and surrogate model observation for all training sets except *x*_*i*_.

The best surrogate model is determined by evaluating performance metrics. The normalized root mean square error (NRMSE) is used as a performance metric. In terms of using *N* as the number of measurement points, $\hat{y_{j}}$ is the surrogate model response at point *j* and *y*_*t*_ is the (mean) experimental value at time t for output *i* at point *j*. The NRMSE is calculated using Equation (27) with respect to the experimental data (*y*_*t*_).

(27) $${NRMSE}_{i}=\frac{\sqrt{\frac{\sum_{j=1}^{N}(y_{t}-\;{\hat{y_{j}})}^{2}}{N}}}{y_{max}-y_{min}}$$

with *y*_*max*_ and *y*_*min*_ the maximum and minimum experimental values.

Furthermore, the computational time for the construction of each of the surrogate models is reported for all stages, including sampling, parameter estimation, and optimization stages.

#### 2.2.3 Surrogate Modeling Implementation

The surrogate models were constructed using the modules of the surrogate models in UQLab,^[^ ^70^ ^]^ which is a MATLAB‐based uncertainty quantification framework. Two wrapper scripts were used; the first, the COMSOL‐UQLab wrapper, to connect and convert data‐formats between COMSOL (via LiveLink) and UQLab‐MATLAB (R2022a, The MathWorks, Inc., Massachusetts, United States). Second, a wrapper script was implemented to construct surrogate models using adaptive sampling sets. Here, the original UQLab modules (Input, active learning reliability, surrogate models) were updated to incorporate the sampling algorithm. The computational time is divided into three phases: the sampling phase to generate training data, the construction and optimization of the surrogate model, and then the parameter estimation of the degradation model based on the surrogate model. The overall simulation time *T*_*sim*_ is given by

(28) $$T_{sim}={T_{BB}}_{N}+T_{SM}+T_{opt}+T_{C}+T_{I}$$

where T_*BB*_ is the time of execution of the black‐box model for N samples, T_*SM*_ is the training time required to construct the surrogate model, T_*opt*_ time required for parameter estimation. T_*C*_ and T_*I*_ are the total time of communication between workflow steps and the idle time of the corresponding computational unit, both of which can be neglected here because they do not impact the overall simulation time.

### 2.3 Design of Computer Experiments (DOEs) for Surrogate Models

The accuracy and performance of surrogate models are significantly impacted by the quality of the training data used to map the input–output relationship of the original model, sample size and the surrogate techniques.^[^ ^25^, ^30^, ^71^, ^72^ ^]^ Within the scope of surrogate models, two types of sampling strategies are implemented: one‐shot and adaptive strategies.^[^ ^73^, ^74^, ^75^ ^]^ One‐shot sampling methods are static, domain‐based and non‐adaptive. These methods are designed for deterministic numerical experiments, in which the same input always yields the same response. Using these methods, a surrogate model is constructed in a single stage with a fixed set of samples. The minimum sample size required is ten times the number of uncertain parameters (10N + 2). These samples are distributed as evenly as possible over the sampling space, without considering prior knowledge of the original model or physical system.^[^ ^30^ ^]^ The main challenge associated with these methods is the unknown number of training points needed to achieve an acceptable accuracy of the constructed model.^[^ ^71^, ^76^ ^]^ On the other hand, adaptive sampling methods construct the surrogate model sequentially. With these strategies, a surrogate model is initially built using a set number of training points. As an initial sample size for training data, ten times the number of the key parameters of the degradation model are selected following the recommendation of Jonas et al.,^[^ ^77^ ^]^ for expensive black‐box models. By incorporating additional data points into the initial training set, the accuracy of the surrogate model is improved. As soon as the required accuracy is achieved the sampling process stops.^[^ ^76^ ^]^ Adaptive sampling algorithms are designed to strategically introduce new training points into areas characterized by high uncertainty in the surrogate model's response.

To study the effect of choosing the sampling strategy on the performance of surrogate models, we tested four one‐shot sampling methods: Monte Carlo sampling (MC), Latin Hypercube sampling (LHS), Sobol sequence sampling (Sobol) and Halton sequence sampling (Halton). A brief comparison is presented in appendix A. These methods were tested across the three models to assess their performance in handling different dimensions of degradation models. Generally, these methods are characterized by their ability to draw random samples from the parametric space. However, the number of samples, the tendency for random clustering and the effectiveness of these methods are influenced by the complexity of the original model and the number of parameters.^[^ ^30^, ^71^, ^75^ ^]^ The comparison between on‐shot sampling methods was performed and implemented using the UQLab input module and COMSOL‐UQLab wrapper scripts.

An adaptive sampling **Algorithm** **1** based on LHS was implemented to locate new samples in regions within the parametric space that were characterized by high uncertainty.^[^ ^78^ ^]^ The characteristics of these regions are determined in accordance with the calculated ε_*LOO*_ of the surrogate models. This algorithm was implemented using a customized script, which used the predefined UQLab‐MATLAB constrained min‐max (CMM) learning function. If adding new sample points does not improve the model's performance, the sample size is considered sufficient according to the predefined stopping criteria, ε_*LOO*_ < θ, with a predefined threshold θ.^[^ ^79^, ^80^ ^]^

Algorithm 1 **An adaptive sampling algorithm for the construction of surrogate models.**

**Table:** 

**Require**: Input parameter vector (X)
**Ensure**: Parameter distribution is uniform distribution $Z∼U{\left[0,1\right]}^{M}$
**Ensure**: Number of key parameters *N*_*p*_ and total sampling set *N*_*T*_
Bonded design space of X as [lower limit, higher limit]
Number of initial sampling set using LHS, *N* = 10*N*_*p*_
Initial sampling set using LHS, *N*_*i*_
Initial experimental $X=x_{1}^{\left(1\right)},\text{…},x_{1}^{\left(N\right)}$
corresponding original model response $Y^{original}=M^{original}\left(X\right)=y_{1}^{\left(1\right)},\text{…},y_{1}^{\left(N\right)}$
Create the surrogate model $M^{SM}$
Set threshold θ = 10^−4^ set threshold
**if** ε_*LOO*_ < θ **then**
*N*_*new*_ = *N*
$M^{S}$ is accepted
**else if** ε_*LOO*_ = θ **then**
minimum accuracy is reached
*N*_*new*_ = *N*
$M^{S}$ is accepted
**else if** ε_*LOO*_ > θ **then**
$N^{*}=\underset{s\in S_{N}}{max}\underset{i=1,\text{…},N}{min}∥N_{T}-N_{i}∥$
find $N^{new}=N\in N_{T}:Y^{SM}\left(N\right)<q$
**end if**

For the parameter estimation of the degradation models, all the parameters are assumed to be uniformly distributed within the testing ranges. Moreover, initial tests using the rate equation and guided by the experimental data are performed to specify the testing ranges for each of the uncertain parameter. The parametric space of the uncertain parameters are constructed for each model using the maximum and minimum limits of the test intervals, these values are given in **Table** **2**.

**Table 2:** The ranges of parameters for the degradation models, and the optimal parameters estimated by Kriging, PCE, and PCK models.

Parameter	Input range	Optimal value			Ref.
		Krg	PCE	PCK	
Model 1 ‐ Equations 11					
k_*deg*_ / mol m^−2^s	−1 · 10^−4^ – 1 · 10^−9^	−2.4321 · 10^−5^	−2.4332 · 10^−5^	−2.4343 · 10^−5^	[39]
*t*_*init*_ /day	0 – 3	1.932	1.933	1.929	
Model 2 ‐ Equations 4 ‐ 9					
*k*_1_ /m^7^ mol^−2^	−1 · 10^−19^ – 1 · 10^−23^	−9.0245 · 10^−21^	−9.0302 · 10^−21^	−9.05301 · 10^−21^	[39]
*k*_2_ /m^7^ mol^−2^	−1 · 10^−5^ – 1 · 10^−12^	−7.0332 · 10^−9^	−7.0502 · 10^−9^	−7.0180 · 10^−9^	
*k*_3_ /m^7^ mol^−2^	−1 · 10^−5^ – 1 · 10^−12^	−7.0023 · 10^−10^	−7.0045 · 10^−10^	−7.0038 · 10^−10^	
*k*_4_ /m^7^ mol^−2^	−1 · 10^−1^ – 1 · 10^−12^	−1.0798 · 10^−2^	−1.096 · 10^−2^	−1.0878 · 10^−2^	
*k*_5_ /m^7^ mol^−2^	−1 · 10^−19^ – 1 · 10^−25^	−8.0037 · 10^−24^	−8.0065 · 10^−24^	−8.0029 · 10^−24^	
*k*_6_ /m^7^ mol^−2^	−1 · 10^15^ – 1 · 10^20^	9.001 · 10^16^	9.01 · 10^16^	9.0024 · 10^16^	
Model 3 ‐ Equations 17					
$D_{Mg^{2+}}$ Mg‐5Gd	1 · 10^−12^ – 1 · 10^−4^	6.05 · 10^−9^	6.125 · 10^−9^	6.053 · 10^−9^	[8]
$D_{Mg^{2+}}$ Mg‐10Gd	1 · 10^−12^ – 1 · 10^−4^	9.53 · 10^−10^	9.721 · 10^−10^	9.608 · 10^−10^	

### 2.4 Uncertainty Quantification Workflow

**Figure 2:**
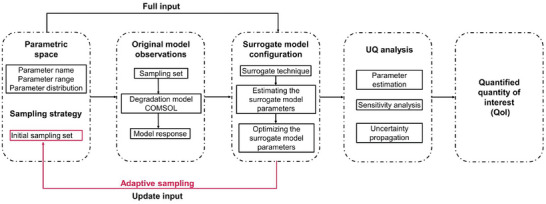
Workflow for quantifying the uncertainty associated with degradation models of Mg‐based biodegradable implants using the surrogate modeling approach.

The following workflow is proposed for conducting UQ analyses for degradation models of Mg‐based biodegradable implants. The workflow is illustrated in **Figure** **2**, where four main steps are presented. The first step involves designing the computer experiments, as detailed in Section 2.3. This step includes defining the parametric space of the model's key parameters, determining sampling strategies and creating the sampling set. It is crucial to define the distribution of the key parameters and to specify the Quantities of Interest (QoI) that need to be quantified, as presented in Table 1 for the three cases under consideration. Additionally, if adaptive sampling is used, the initial sampling set $X_{train,init}$ must be defined in this step. Moving on to the second step of the workflow, which is how to deal with the original models. At this step, observations $Y_{train,init}$ of the original models are gathered by evaluating the degradation models for the sampling sets generated during the DOE step. The construction of a surrogate model $M^{S}$ (Equation 21) is the focus of the third step. Here, the surrogate model functions are mapped onto the user‐defined parametric space using observations from the original model. The surrogate models are trained and their parameters are optimized. In the case of adaptive sampling, this step will involve iteratively training the surrogate model until achieving the best surrogate model with the lowest (ε_*LOO*_) (Equation 26). The optimal surrogate model, whether it be Kriging, PCE or PCK, is subsequently used for further UQ analysis. These analyses include parameter estimation, uncertainty propagation and sensitivity analysis. As the UQ analysis progresses, the original models are characterized, leading to the quantification of QoIs. As a result, the uncertainties associated with these quantities are taken into account during their presentation.

#### 2.4.1 Sensitivity Analysis

Sobol' sensitivity analyses were performed to evaluate the variance contribution of each individual and combination of key parameters to the variance of the degradation model output.^[^ ^81^, ^82^, ^83^ ^]^ Throughout this study, how the variation in k_*deg*_ and t_*init*_ affects the degradation of pure Mg was examined, whereas the effect of varying k_*deg*_, t_*init*_ and k_*i*_ on the precipitation model response is tested. Results for the variance‐based sensitivity analysis are expressed by two sensitivity indices: the Sobol' first order sensitivity indices *S*_*i*_, Equation (29a), indicate the importance of each parameter considered individually, while the total sensitivity indices $S_{T_{i}}$, Equation (29b), account for both the importance of individual parameters and interactions between parameter pairs.^[^ ^81^ ^]^

(29a) $$\begin{array}{c} S_{i}=\frac{Var(M_{i}\left(X_{i}\right))}{Var(M(X))} \end{array}$$

(29b) $$\begin{array}{c} S_{k}^{T}=\sum_{k\in u}S_{u}\;\text{for}\;\;k=\left\{1,2,\text{…},P\right\} \end{array}$$

where *X* is the parameter vector, *X*_*i*_ is a subset of surrogate model inputs with corresponding outputs *M*_*i*_ and *P* is the number of surrogate model evaluations. *M*_*i*_ is the surrogate model shape function defined by Equation (21).

#### 2.4.2 Propagation of Uncertainty

Uncertainty propagation is the process of predicting the influence of uncertainties (e.g., measurement errors, model defects, etc.) in input variables on the uncertainty of output variables in a system or process,^[^ ^84^, ^85^ ^]^ which can then be utilized to improve decision‐making and risk assessment. To assess the propagation of uncertainty within the surrogate models the variation in the input parameters was reported in terms of ε_*LOO*_ (Equation 26). Here, we tested the change in ε_*LOO*_ between the single‐shot and adaptive sampling. Moreover, the effect of changing the number of uncertain parameters over the surrogate models was also reported.

## 3 Results and Discussion

### 3.1 The Construction of Surrogate Models

**Figure 3:**
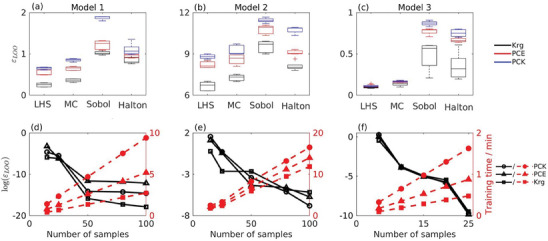
DOE comparison across the three degradation models, each represented by a column. Row 1 (panels a–c) illustrates the effect of different sampling methods on the ε_*LOO*_ error across the surrogate models. Row 2 (panels d–f) depicts the number of adaptive samples obtained using Algorithm 1, as functions of accuracy (ε_*LOO*_), training time, and the type of surrogate model.

The four different sampling methods tested in this study compared in terms of the average ε_*LOO*_ values of the constructed surrogate models based on ten random one‐shot sample runs. The construction of each surrogate model was performed using the same test samples. Therefore, for the testing purpose of the single‐shot sampling methods, it was necessary to gather ten sample sets, with each set containing 22, 82, and 12 samples for models 1, 2, and 3, respectively. For single‐shot sampling, **Figure** **3**a–c shows that surrogate models constructed with training data sampled using LHS have the lowest ε_*LOO*_ values for all degradation models. Furthermore, LHS reduces the number of samples required while maintaining inference accuracy, with a conversion error given by *O*((*i* − 1)/*N*, *i*/*N*), where N represent the equally probable intervals between each input variable, and 𝑖 represent a variable corresponding to one of these intervals, where i ∈ [1,N]. This can be attributed to the space‐filling nature of LHS.^[^ ^74^ ^]^ In general, LHS generates random samples by dividing the range of input variables into equally probable intervals. In each dimension, each interval is sampled only once, which helps prevent random clustering issues as is the case with MC sampling. Nonetheless, surrogate models constructed using MC sampling have ε_*LOO*_ values close to LHS, as can be seen for model 1 and model 2, Figure 3a,b, respectively.

As the number of uncertain parameters increases, the degradation model becomes increasingly complex. This is illustrated in Figure 3b for model 2. Using single‐shot sampling, more data sets are needed to enhance the accuracy of the constructed surrogate models for model 2. Moreover, the relatively high ε_*LOO*_ values for all single‐shot sampling strategies presented in Figure 3a–c suggest that these strategies were unsuccessful in producing representative data sets that could be used to construct surrogate models with sufficient accuracy. Both Sobol and Halton sampling exhibit relatively high ε_*LOO*_ due to the strong correlation between generated samples resulting from the low dimensionality of the parametric space (*d*) of the three degradation models (1 ⩽ *d* ⩽ 10).^[^ ^22^ ^]^ Low dimensionality of the degradation model results in higher variation in ε_*LOO*_ values as can be observed for model 1, *d* = 2 and model 3, *d* = 1. While, for model 2, which has *d* = 8 less variation is observed. However, a large number of samples is necessary to ensure convergence due to the dependency of error conversion on both number of samples and dimensionality, namely *O*(*log*(*N*)^*d*^/*N*) for Halton sampling, while it is *O*(*log*(*N*)^*d*^/*N*) for Sobol sampling.

In general, as the dimensionality of the degradation model increases more samples are required to construct the surrogate model.^[^ ^30^, ^80^ ^]^ As shown in Figure 3c, using the same sampling set constructed with LHS results in the same accuracy for all surrogate models of Model 3 with d = 1. This is because the dimensionality of a surrogate model is determined by the number of input parameters it needs to represent, which, in this case, is the diffusion coefficient of Mg^2+^ ions. For model 1 and 2 both PCE and PCK require more samples to improve their convergence and reduce ε_*LOO*_ values. On the other hand, Kriging models outperform the other surrogate models due to their ability to handle limited data sets since they were designed to utilize minimal sampling. This can be explained by the fact that the Kriging‐based technique interpolates the simulation results and fits the Maximum Likelihood Estimation as well as the unbiased Linear Predictor‐criterion on the results. Hence, it is recommended to use Kriging for cases where the dimensionality is less than 20.^[^ ^30^, ^80^ ^]^

The problem of training data size arises as the number of original model parameters increases, leading to increase the number of required samples. As evident from the results of one‐shot sampling, as the size of the sampling set influence the cost associated with training and constructing these models. Figure 3(d–f) summarizes the ability of adaptive sampling algorithm based on LHS and using the semi‐supervised CMM learning function on optimizing the number of samples, based on function of accuracy (ε_*LOO*_) and training time to construct the surrogate models for the three degradation models. It is clear that as the sample size increases the training time increases for all surrogate models. Furthermore, the implementation of the adaptive sampling approach indicates that constructing surrogate model with an accuracy of 10^−3^ in terms of ε_*LOO*_ is possible with fewer number of samples even for model 2, which contains 8 uncertain parameters.

**Figure 4:**
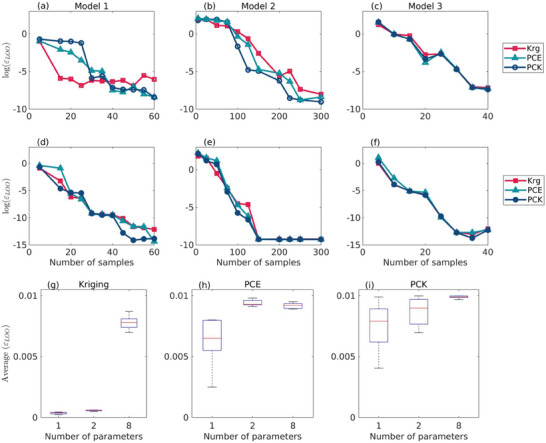
The effect of changing the number of samples and the number of parameters on error propagation. Row 1, panels a–c, illustrate the propagation of error as a function of the number of samples using single‐shot sampling (LHS). Row 2, panels d–f, illustrate the propagation of error as a function of the number of samples using adaptive sampling (Algorithm 1). Row 3, panels g–i, present box plots of the average ε_*LOO*_ for surrogate models as a function of the number of parameters: g) Kriging, h) PCE, and i) PCK.

Adaptive sampling decreases the number of samples required to construct the surrogate models without affecting the accuracy of the models, as can be seen for the three degradation models in **Figure** **4**a,b. The subplot in Figure 4a shows that for single‐shot sampling with LHS, the curves of Kriging and PCE tend to coincide as the number of samples is above 30, while 58 samples are needed for PCK curve to converge to the same value. For adaptive sampling algorithm, Figure 4d, the three curves are coinciding after only 15 samples, which drastically reduces the computational cost and time. A comparable trend is observed for the other degradation models presented in the subplots in Figure 4b,e and c,f. For model 3, panels c, and f all surrogate curves coincide after ten samples. Overall, as the number of samples increases both single‐shot LHS and adaptive sampling algorithm are converging to constant ε_*LOO*_ values. The effect of the number of parameters is presented in the third row of Figure 4g–i. Overall, the implementation of adaptive sampling algorithm reduces the propagation of error within the surrogate models. However, it is clear that for low dimensional models, specifically model 1 with *d* < 3, both PCE and PCK propagate higher error, while Kriging is more efficient due to its ability to deal with small data sets and lower dimensionality. However, for the case with 8 uncertain parameters, model 2, the propagation of error within Kriging is higher with respect to other surrogates due to the tendency of Kriging to form ill‐conditioned matrices under high dimensional models.^[^ ^86^, ^87^ ^]^ In general, as the number of uncertain parameters increases the propagation of uncertainty, in terms of ε_*LOO*_ values, within the models increases too.

**Figure 5:**
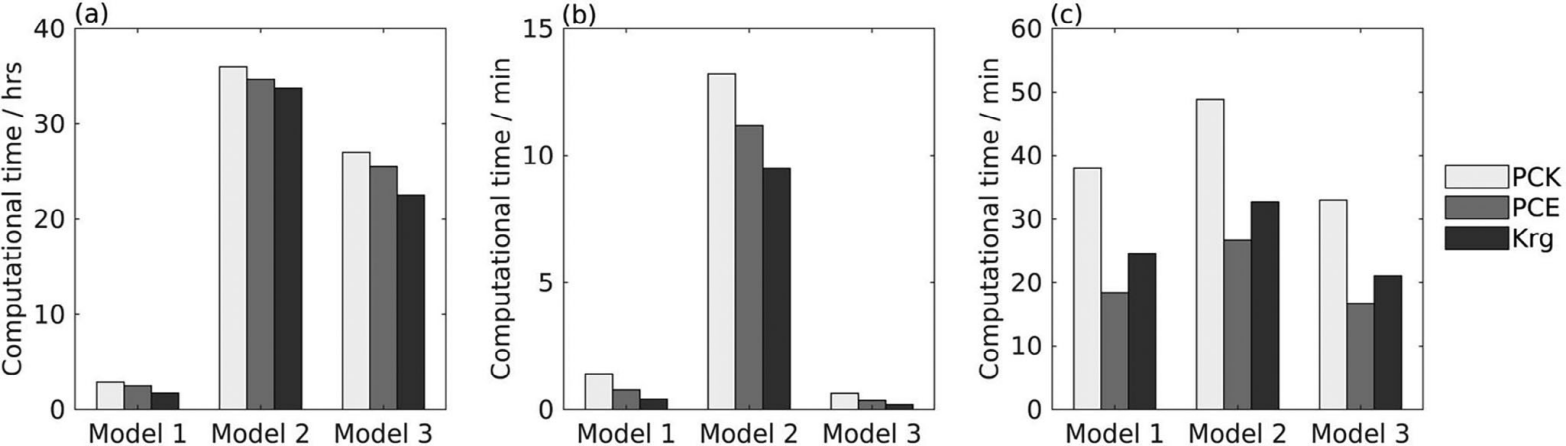
CPU‐time for the three degradation models, a)$T_{BB_{N}}$: total sampling time b) *T*_*SM*_: time to construct a surrogate model, c) *T*_*opt*_: time to perform the parameter estimation based on surrogate models.

**Figure** **5** illustrates the breakdown of CPU‐time. Collecting the training data, or the sampling phase, is the most CPU‐intensive as illustrated in Figure 5a. We anticipate and attribute the extended duration of the sampling stage to the need to execute the original degradation models, which take, on average, 8, 25, and 90 min for each run for each of the three degradation models, respectively. Figure 5a shows that adaptive sampling reduces the CPU‐time for the sampling stage, which is $T_{BB_{N}}$ in Equation (28). Overall, adaptive sampling reduces the $T_{BB_{N}}$ for the three degradation models. For model 2, it $T_{BB_{N}}$ takes twice as long as adaptive sampling, including the initial sampling and searching for new samples. In this case the higher $T_{BB_{N}}$ is attributed to the increased number of uncertain parameters. The CPU‐time for constructing surrogate models, *T*_*SM*_ presented in Figure 5b showed that for the three degradation models PCK required higher time, which contributed to the fact that PCK involves the integration of two different techniques; PCE and Kriging. *T*_*SM*_ for the first and third FE degradation models are less than 1 min and this is due to the low number of uncertain parameters. On the other hand, *T*_*SM*_ increases for model 2 due to the complexity of the degradation model itself and the increased number of uncertain parameters. Figure 5c illustrates the parameter estimation time, *T*_*opt*_, which is required by each surrogate model to estimate the key parameters for each of the degradation models. At this stage, surrogate models are used to speed up the calibration process. Figure 5c highlights that Kriging demands higher *T*_*opt*_ primarily due to the need for estimating the hyperparameters of Kriging. Further, Kriging's determination of the spatial covariance structure of the sampled points contributes to the increased *T*_*opt*_. The ability to analytically compute PCE coefficients significantly contributes to reducing the optimization time for PCE models across all cases,^[^ ^72^ ^]^ which also contributes to decreasing the computational costs of PCK.

In summary, the choice of sampling strategy and the size of the training sample set are critical for constructing accurate, robust, and efficient surrogate models, as well as for overcoming issues of underfitting. The findings underscore that as the dimensionality of the degradation model increases (model 3 < model 1 < model 2), a larger number of samples is necessary for accurate surrogate model construction. However, the proposed adaptive sampling algorithm reduced the required number of samples without compromising accuracy. Furthermore, PCE and PCK demonstrated lower error propagation in models with dimensions greater than two (model 2). CPU time analysis revealed that adaptive sampling significantly reduced the CPU time required for the sampling phase, particularly in high‐dimensional models. PCE had the lowest time required to construct the surrogate models, while Kriging required more time for parameter estimation due to hyperparameter (*kernel*) optimization, and PCK had higher model construction times due to its integration of PCE and Kriging techniques.

To construct the surrogate models, initially, five sets of samples for each key parameter were randomly generated using LHS method and refined according to the adaptive sampling Algorithm 1. In total, three sets of sample groups were generated for each surrogate model. Kriging required 15, 50, and 7 samples for the three respective FE degradation models. For PCE and PCK, 25 samples were needed for models 1 and 2, while 10 samples were generated for model 3.

### 3.2 Performance of the Different Surrogate Models

Tested ranges for the key parameters of the degradation models and the optimal parameters as estimated by Kriging, PCE, and PCK for each model are given in Table 2. The key parameters estimated by the three surrogate models for all the degradation models have relatively close values. Based on this observation and considering the critical characteristics of size, time and accuracy, Kriging models are more suitable for parameter estimation tasks requiring lower computational time and higher accuracy. This observation is supported by the small reduction in the ε_*LOO*_ for the three surrogate models as presented in **Table** **3**, for example for model 1, ε_*LOO*_ value, based on adaptive sampling, is 1.296 · 10^−4^ for PCE and 1.308 · 10^−5^ for PCK compared to 2.03 · 10^−4^ for Kriging.

**Table 3:** Validation of the performance of three surrogate models at the optimized number of samples, based on adaptive sampling, for degradation models. The performance is evaluated in terms of ε_*LOO*_ for surrogate model accuracy and NRMSE with respect to experimental data.

Model	ε_*LOO*_			NRMSE		
	Kriging	PCE	PCK	Kriging	PCE	PCK
Model 1						
MMD	2.03 · 10^−4^	1.296 · 10^−4^	1.308 · 10^−5^	0.07	0.05	0.051
Model 2						
Mg	5.312 · 10^−4^	3.703 · 10^−5^	1.082 · 10^−5^	0.99	1.003	1.006
Ca				0.53	0.40	0.62
C				0.59	0.56	0.60
P				0.38	0.36	0.362
O				1.03	0.98	1.01
Model 3						
VL%_*Mg*−5*Gd*_	3.01 · 10^−4^	2.13 · 10^−6^	4.861 · 10^−6^	0.03	0.07	0.08
VL%_*Mg*−10*Gd*_	7.04 · 10^−4^	3.71 · 10^−6^	6.089 · 10^−6^	0.02	0.08	0.09

At each step, the performance of the surrogate models is evaluated in terms of ε_*LOO*_, which measures the accuracy of the predictions of the surrogate model. During this stage, the key parameters outlined in Table 2, along with their respective ranges for constructing the parametric space, are estimated and quantified using surrogate models. The agreement between surrogate and original models are visualized in the QQ‐plots in Appendix B. All the points on the QQ‐plots of all surrogate models and tested degradation models fall near the 45‐degree line, which indicates that the surrogate models are capturing the same underlying patterns in the data as the original degradation model.^[^ ^88^, ^89^ ^]^

Subsequently, the optimal values of the key parameters are used to calibrate the output of the degradation models with respect to the in‐house experimental data (Section. 2.1.2).

**Figure 6:**
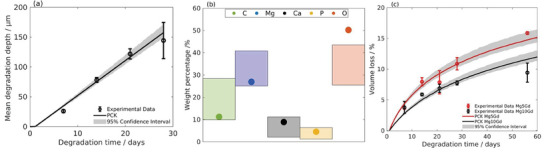
The performance of PCK for the three degradation models: a) model 1, b) model 2, the weight percentage plot, the scatter points are the predicted weight percentage from the PCK model, while the shaded areas indicate the minimum and maximum mean values from the experiment c) model 3.

In view of the NRMSE values provided in Table 3, small deviation in the results can be observed for the performance of the surrogate models with respect to experimental data. This difference is due to the mathematical nature of the surrogate models, which controls the creation of the surrogate mapping function, explained in Table 1. The different mapping function of surrogate models enables Kriging to interpolate local variations of the output of the degradation model. Polynomial surrogates (e.g., PCE), on the other hand, are generally approximated by the global behaviors of degradation models. In this way, PCK can explain the better agreement with experimental data, as it combines local and global approximations. However, the efficiency of the adaptive‐sampling leads to enhance the quality of the training data, hence, the surrogate models, Kriging, PCE and PCK are able to characterize the three degradation models accurately, as can be seen in **Figure** **6** for PCK model. The performance of PCE and Kriging for the three degradation models figures are presented in Appendix B. However, it is crucial to note that the generalization of any of these constructed surrogate models to simulate other implants under different conditions or degradation phenomena necessitates further validation and training.

All surrogate models performed robustly and captured the underlying data patterns, with PCK offering the best agreement by incorporate both interpolation of local variations (from Kriging) and approximate the global behaviors of degradation models by the polynomial trend (PCE). However, Kriging demonstrated slightly higher computational efficiency (less CPU time) with acceptable accuracy (based on NRMSE) for parameter estimation.

### 3.3 Sensitivity Analysis (SA)

**Figure 7:**
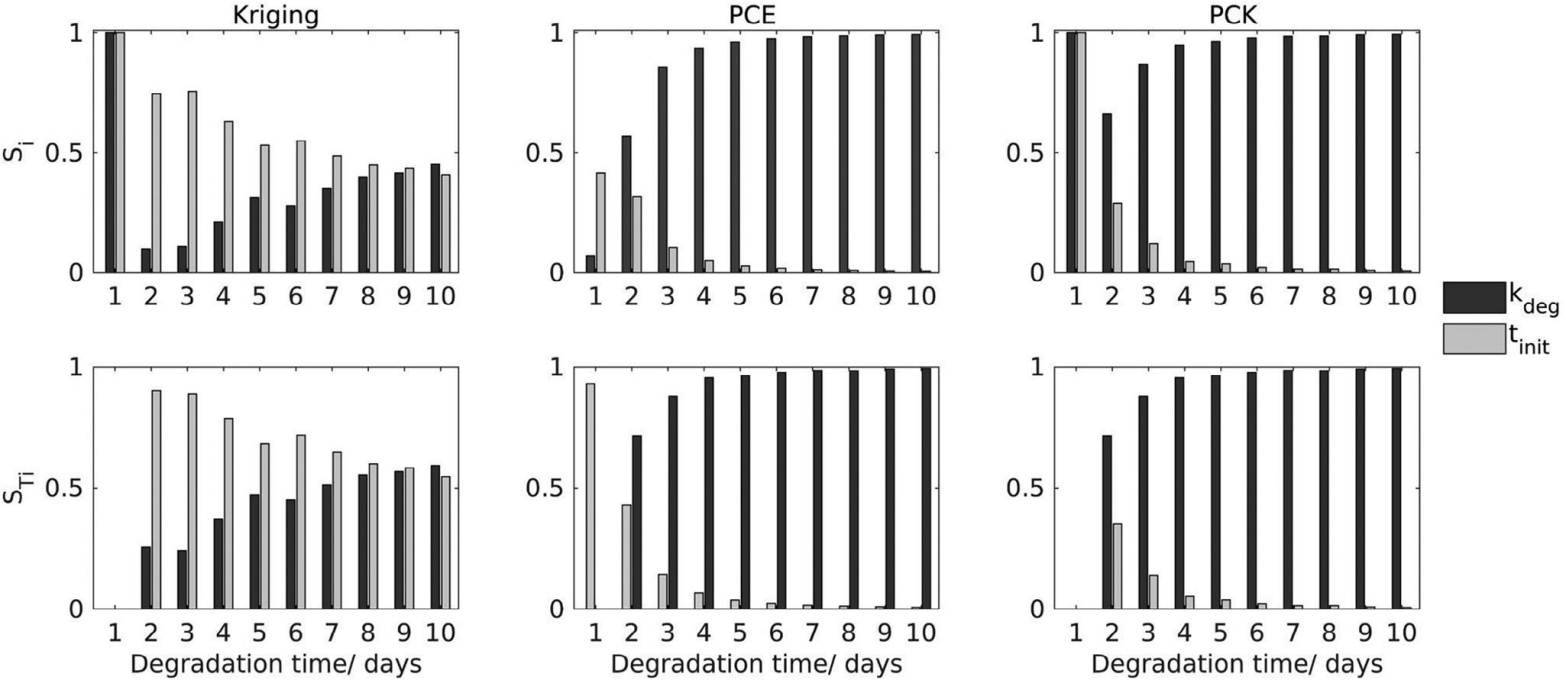
Global sensitivity analysis of case 1 for the first ten days of degradation. The Sobol' indices (for uncertain parameters of the degradation model. The first Sobol' indices (*S*_*i*_) and total effect indices ($S_{T_{i}}$) measured following surrogate‐based global sensitivity analysis for the degradation reaction, where the uncertain input variables are k_*deg*_ and t_*init*_ and the QoI is MDD.

To assess the sensitivity of model responses to uncertainties in input parameters, Sobol' indices were computed and compared for the multi‐parameter models (models 1 and 2). The first‐order Sobol'  indices ($S_{i}$) quantify the direct effect of each individual parameter, while the total Sobol' indices ($S_{T_{i}}$) capture all interactions involving that parameter. For clarity, the Sobol' indices for the three surrogate models constructed for the degradation model 1 are presented in a bar chart in **Figure** **7**. The indices were visualized for the first ten days of degradation, as the values stabilized beyond this point. For the Kriging surrogate model of model 1, S_*i*_ indicates that the two parameters k_*deg*_ and t_*init*_ primarily compete during the first ten days, with t_*init*_ being the most influential over MDD. After the eighth day, both parameters exert nearly equal influence. This pattern is mirrored in the total Sobol'  indices ($S_{T_{i}}$). In contrast, the Sobol' indices derived from the PCE and PCK models suggest that t_*init*_, mainly affects the initial sigmoid activation during the early stages (2 days) of the degradation process, after which k_*deg*_, becomes dominant as the degradation process is governed by the degradation reaction. This finding aligns with the mathematical expression of MDD (Equation 11). Moreover, the small differences between S_*i*_ and $S_{T_{i}}$ suggest that the interaction between k_*deg*_ and t_*init*_ is relatively minor on the long term and can be disregarded without significantly affecting the model's predictions if a proper optimization step is introduced for t_init_. Consequently, the model complexity can be reduced by running the model for only up to three days and optimizing and setting a constant value for *t_*init*_*. With the simplified model, the other parameters can then be calibrated for. However, the Sobol' indices based on the Kriging surrogate model suggest that both parameters have relatively equal influence over MDD, indicating a correlation between them. This finding appears contradictory, given that the negligible difference between S_*i*_ and $S_{T_{i}}$, except on day 1, supports the same conclusion as the PCE and PCK models: there is no significant interaction between these two parameters. The observed differences in the interpretation of Sobol' indices across surrogate models can be attributed to the methods used to compute these indices. In the case of PCE and PCK, the Sobol' indices are calculated analytically by evaluating the integrals in the model decomposition, which significantly reduces computational costs. On the other hand, Kriging struggles to reflect these interactions due to the stochastic uncertainty introduced by the Monte Carlo methods typically used to evaluate Sobol' indices. This discrepancy highlights the importance of clearly defining the objectives of using surrogate models, i.e., whether they are applied only to accelerate tasks such as parameter calibration, or whether they are used for understanding the underlying system and the influence of certain parameters thereon as in sensitivity analysis.

**Figure 8:**
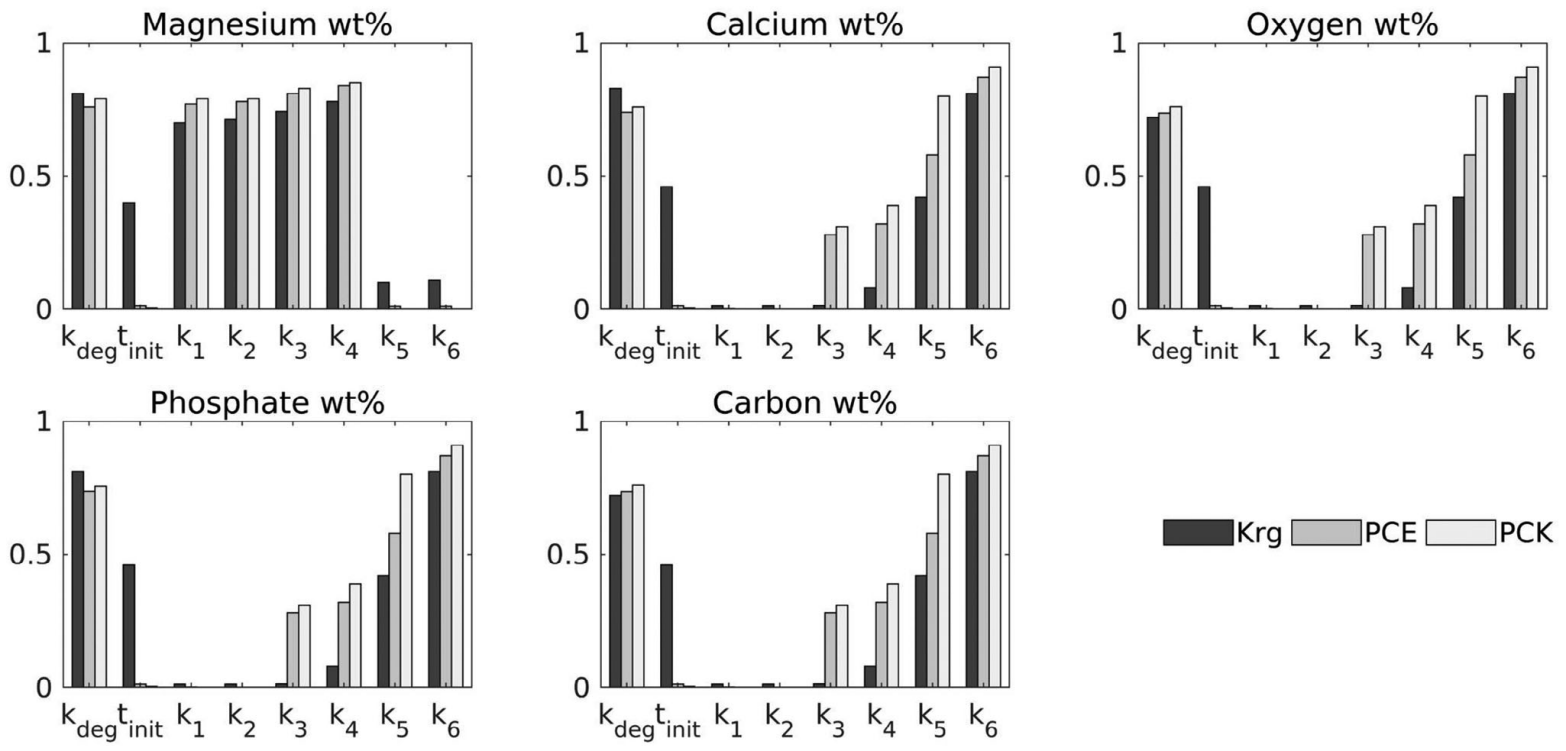
Sobol' indices ($S_{T_{i}}$) for key parameters of precipitate formation on the surface of Mg implant model, a) Oxygen, b) Magnesium c) Carbon, d) Calcium, and e) Phosphate. The total effect indices ($S_{T_{i}}$) were measured following surrogate‐based global sensitivity analysis. The sum of ($S_{T_{i}}$) can exceed one. In the absence of interactions, equality is obtained.

For model 2, which simulates the precipitation reactions during Mg degradation, the calculated Sobol' indices were found to be nearly equal ($S_{T_{i}}-S_{i}<0.005$) for all surrogate models, so only $S_{T_{i}}$ are presented in **Figure** **8**. The sensitivity analysis showed similar results between the PCE and PCK models, but significant differences were observed when compared to the Sobol' indices derived from the Kriging surrogate. Specifically, when assessing the influence of the degradation kinetic parameters, k_*deg*_ and t_*init*_, on the wt% of Mg, Ca, O, P, and C, the Kriging model indicated that t_*init*_ has a significant effect. In contrast, both PCE and PCK showed that t_*init*_ has a negligible impact, which aligns with the formulation of model 2 where t_*init*_ had limited influence on precipitate formation in the degradation layer. Thus, t_*init*_ can be excluded from further analysis and assigned a constant value to reduce the dimensionality of the analysis. Meanwhile, k_*deg*_ is found to influence the formation of all precipitates, which means it is crucial to include the kinetic of the degradation during the analysis of the precipitation process. All three surrogate models consistently indicated that parameters k_1_ and k_2_ have a minimal effect on wt% of Ca, O, P, and C, suggesting that these parameters can be treated as constants for modeling these elements. This is surprising, as both brucite (Equation 4, k_1) and magnesite (Equation 5, k_2) contain O and the latter also C. In contrast, the models revealed a significant influence of k_1_ and k_2_ on wt% of Mg, indicating that calibration for both parameters should focus primarily on the wt% of Mg. Interestingly, k_3_ and k_4_ had uniform effects over all elemental weight percentages except Mg, which suggests that both parameters might be considered as global parameters that affect all elemental wt% equally except wt% of Mg. The exception for Mg in relation to k_3_ and k_4_ likely stems from correlations between precipitate reactions. Moreover, except for Mg, all elemental wt% are strongly influenced by k_5_ and k_6_, which are mainly associated with the precipitation of two precipitation calcite and hydroxyapatite. The strong correlation shown by $S_{T_{i}}$ indicates the presence of competing reactions between calcium and phosphate ions in the formation of hydroxyapatite. Furthermore, similar dependencies were observed for oxygen, magnesium, and carbon. These findings not only streamline the modeling process but also highlight potential mechanistic differences in elemental behaviors, particularly for Mg, offering directions for further investigation into the system's underlying chemistry. Note that both PCE and PCK effectively captured these correlations, whereas the Sobol' indices derived from Kriging did not reflect all the interactions. This disparity may be due to the tendency of Sobol' indices derived from Kriging to emphasize underlying global trends in the data samples. Additionally, the mathematical assumption of a constant mean (Equation 23) limits the precision of the Kriging models' predictive ability.

The Sobol' indices derived based on PCE and PCK reflected the influence of the key parameters and their interactions on the degradation models more accurately than Kriging. The results of sensitivity analysis not only streamline the modeling process, but also suggest avenues for further research into the mechanisms governing the elemental behaviors in the system.

## 4 Conclusion

This research paper presents a comprehensive Uncertainty Quantification (UQ) workflow, designed to enhance the development and quantification of mathematical models simulating the biodegradation of magnesium‐based implants. Key findings include:

1. Surrogate model selection is crucial: Kriging excels in parameter estimation, while PCE and PCK are superior for uncertainty prediction, particularly in high‐dimensional systems.
2. Adaptive sampling significantly reduces required sample sizes without compromising accuracy (ε_*LOO*_ < 10^−4^), addressing the computational challenges of UQ analysis.
3. The workflow demonstrates high adaptability across various degradation systems and model complexities, providing a robust framework for UQ implementation.
4. Rigorous validation of surrogate models is essential to maintain system accuracy and reliability.

The developed UQ workflow offers a scalable and efficient approach for quantifying uncertainty in Mg‐based implant degradation models. While future work may focus on more complex in vivo corrosion scenarios, this study establishes a solid foundation for surrogate modeling in simpler in vitro models. The methods presented are well‐established and flexible, making them applicable to a wide range of corrosion and degradation models, regardless of complexity. This research significantly contributes to the field by providing a best‐practice guide for implementing UQ in biodegradation modeling, paving the way for more accurate and computationally feasible analyses in biomedical engineering and related disciplines.

Moreover, future work could focus on applying the proposed UQ workflow to a wider range of stochastic and phenomenological degradation models. Additionally, it would be valuable to investigate the performance of surrogate models in capturing multi‐scale interactions in complex degradation processes, as well as exploring the integration of domain‐specific knowledge to enhance surrogate model accuracy for specialized degradation phenomena.

## Conflict of Interest

The authors declare no conflict of interest.

## Author Contributions

T.A. contributed to various key aspects of the project, including conceptualization, formal analysis, software development, methodology, investigation, and the writing of the original draft. R.W.R. played a supervisory role while also providing valuable insights through writing review and editing. Meanwhile, B.Z.P. focused on supervision and secured funding for the project, in addition to contributing to conceptualization, editing and writing of the original draft.

## Figures and Tables

**Figure A1:**
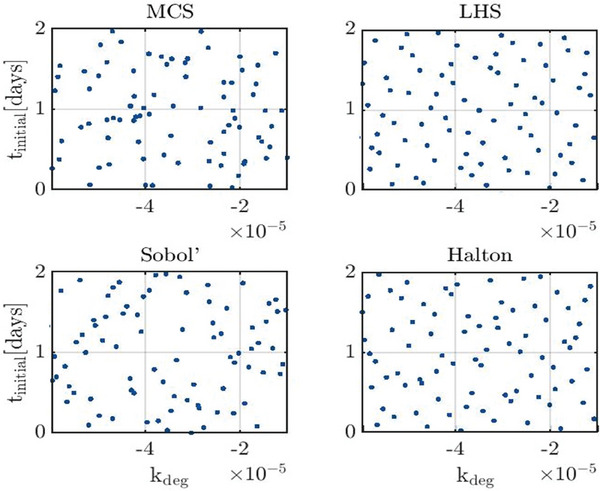
Samples generated by various sampling scheme of the two input parameters; *t*_*init*_ and *k*_*deg*_. The parameters are plotted against each other in pairs, using different sampling schemes: Monti Carlo sampling (MCS), Latin hypercube sampling (LHS), sampling based on Sobol' sequences and Halton sampling. Each of the parameters follows independent a uniform distribution within the range [03] for *t*_*init*_ and [− 1×10^−4^ – 1×10^−9^] for *k*_*deg*._

**Figure B1:**
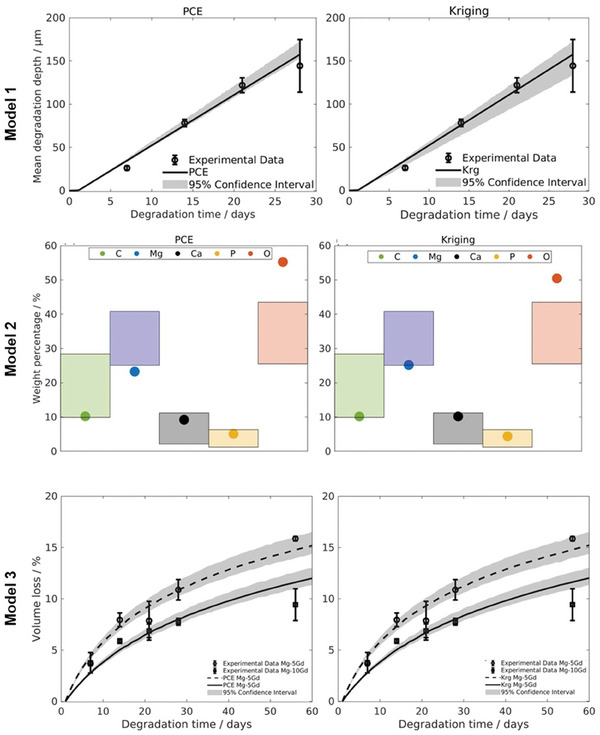
The performance of PCE and Kriging for the three degradation models.

**Figure B2:**
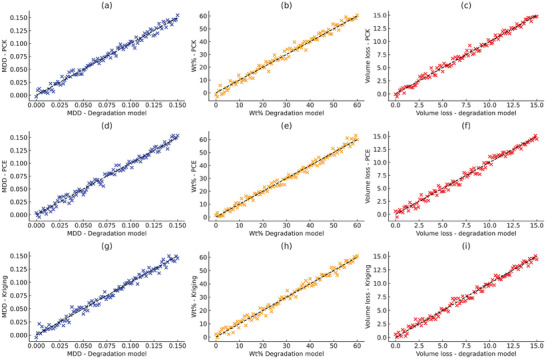
QQ plots for the three degradation models, columns are model 1, model 2 and model 3, row 1 is QQ plot to compare degradation models predictions versus PCK, row 1 is QQ plot to compare degradation models' predictions versus PCE and row 3 row 1 is QQ plot to compare degradation models predictions versus Kriging.

## Data Availability

The data that support the findings of this study are available from the corresponding authors upon reasonable request.

## Appendix A Brief Review of the Sampling Strategies

For the scope of the current work, we compared four different sampling strategies Monte Carlo sampling (MC), Latin Hypercube sampling (LHS), Sobol' sequence sampling (Sobol) and Halton sequence sampling (Halton). Statistically, these models are good choices because of their one‐shot sampling characteristics, which allows them to determine the sample size at once. The main problem associated with these methods is the possibility of under‐ or over‐sampling.^[^ ^25^ ^]^ MC sampling is a non‐intrusive method, no changes to the model are required, and it is a straight forward method, where random samples are drawn from the probability distribution of the input and then run it through the test models. Slow conversion is one of the major drawbacks of MC, where the sampling error decreases as $O(1/\sqrt{N})$. For a half‐reduction in sampling error, the sample size needs to be quadrupled.^[^ ^90^ ^]^ Nonetheless, for UQ tasks with high dimensionality, MC is still a competitive method because it is not affected by the dimensionality of inputs, which does not affect sampling error.^[^ ^91^ ^]^ By using Latin Hypercube sampling (LHS), the number of samples is reduced while maintaining inference accuracy with a sampling error *O*((*i* − 1)/*N*, *i*/*N*). LHS generates random samples by dividing the ranges of the input variables into equally probable intervals, where each interval in each dimension is only sampled for one time, which avoid the random clumping that can occur with pure MC, as seen in **Figure** **A1**. Due to this LHS has better space‐filling properties compared to other sampling methods.^[^ ^65^, ^90^, ^92^ ^]^

There is no dimensional limitation to LHS, which is an advantage over the Halton sampling *O*(*log*(*N*)^*d*^/*N*). Adding to that, Helton sampling is a deterministic method that works for *d* > 1 and tends to generate samples with strong correlations for lower dimensions.^[^ ^22^ ^]^ Sobol sampling claimed to be superior to MC and LHS but to reduce the sampling error and ensure convergence a non‐realistic number of samples is required (above 1 million).^[^ ^22^, ^90^ ^]^ Considering the previous discussion, for the biodegradation system for Mg‐based implants we choose to use LHS, which is able to produce more consistent results with fewer number of runs across different dimensions of the models.^[^ ^65^, ^90^, ^92^ ^]^ Furthermore, the use of LHS is more advantageous for models with high input, such as the formation of precipitates on a Mg surface model. This is a higher dimensional model with eight input parameters. The increased number of samples can be added to the experiment design through sample enrichment. This is where additional samples are generated based on the analysis of the previous samples via nested LHS, where the enriched samples formed a pseudo LHS.^[^ ^93^ ^]^

## Appendix B The Performance of Surrogate Models

The **Figure** **B1** shows the performance of the surrogate models for the three degradation models estimated using PCE and Kriging, while **Figure** **B2** shows the QQ‐plots, which compare the predictions of the degradation models versus the surrogate models.
